# Supplementary material for: MetaQTL: a package of new computational methods for the meta-analysis of QTL mapping experiments
Source: BMC Bioinformatics. 2007 Feb 8;8:49. doi: 10.1186/1471-2105-8-49 (PMC1808479; doi:10.1186/1471-2105-8-49)
Supplement: Additional File 7 — MetaQTL Package : jar file and tutorial. This Zip archive contains both the MetaQTL JAR file and the files of the tutorial. [file 1471-2105-8-49-S7.zip › org.inra.metaqtl/doc/org/metaqtl/graph/ChromLayer.CMrkTickPoint.html]

ChromLayer.CMrkTickPoint


|  |  |  |
| --- | --- | --- |
| **Overview** \| **Package** \| **Class** \| **Use** \| **Tree** \| **Deprecated** \| **Index** \| **Help** | |  |
| **PREV CLASS**   **NEXT CLASS** | **FRAMES**    **NO FRAMES**     **All Classes** |  |
| SUMMARY: NESTED \| FIELD \| CONSTR \| METHOD | DETAIL: FIELD \| CONSTR \| METHOD |  |


---


## org.metaqtl.graph Class ChromLayer.CMrkTickPoint

```
java.lang.Object
  org.metaqtl.graph.ChromLayer.CMrkTickPoint
```

**Enclosing class:**: ChromLayer

---

``` public class ChromLayer.CMrkTickPoint extends java.lang.Object ```

---

| **Field Summary** | |
| --- | --- |
| `(package private)  java.awt.geom.Point2D` | `p1` |
| `(package private)  java.awt.geom.Point2D` | `p2` |
| `(package private)  int` | `status` |


**Constructor Summary**

`ChromLayer.CMrkTickPoint()`


**Method Summary**

**Methods inherited from class java.lang.Object**

`clone, equals, finalize, getClass, hashCode, notify, notifyAll, toString, wait, wait, wait`

**Field Detail**

### p1

```
java.awt.geom.Point2D p1
```

---


### p2

```
java.awt.geom.Point2D p2
```

---


### status

```
int status
```


**Constructor Detail**

### ChromLayer.CMrkTickPoint

```
public ChromLayer.CMrkTickPoint()
```


---


|  |  |  |
| --- | --- | --- |
| **Overview** \| **Package** \| **Class** \| **Use** \| **Tree** \| **Deprecated** \| **Index** \| **Help** | |  |
| **PREV CLASS**   **NEXT CLASS** | **FRAMES**    **NO FRAMES**     **All Classes** |  |
| SUMMARY: NESTED \| FIELD \| CONSTR \| METHOD | DETAIL: FIELD \| CONSTR \| METHOD |  |


---
